# Supplementary material for: Prolonged exposure to lung-derived cytokines is associated with activation of microglia in patients with COVID-19
Source: JCI Insight. 2024 Mar 19;9(8):e178859. doi: 10.1172/jci.insight.178859 (PMC11141878; doi:10.1172/jci.insight.178859)
Supplement: Supplemental data [file jciinsight-9-178859-s041.pdf]

Supplemental Figures

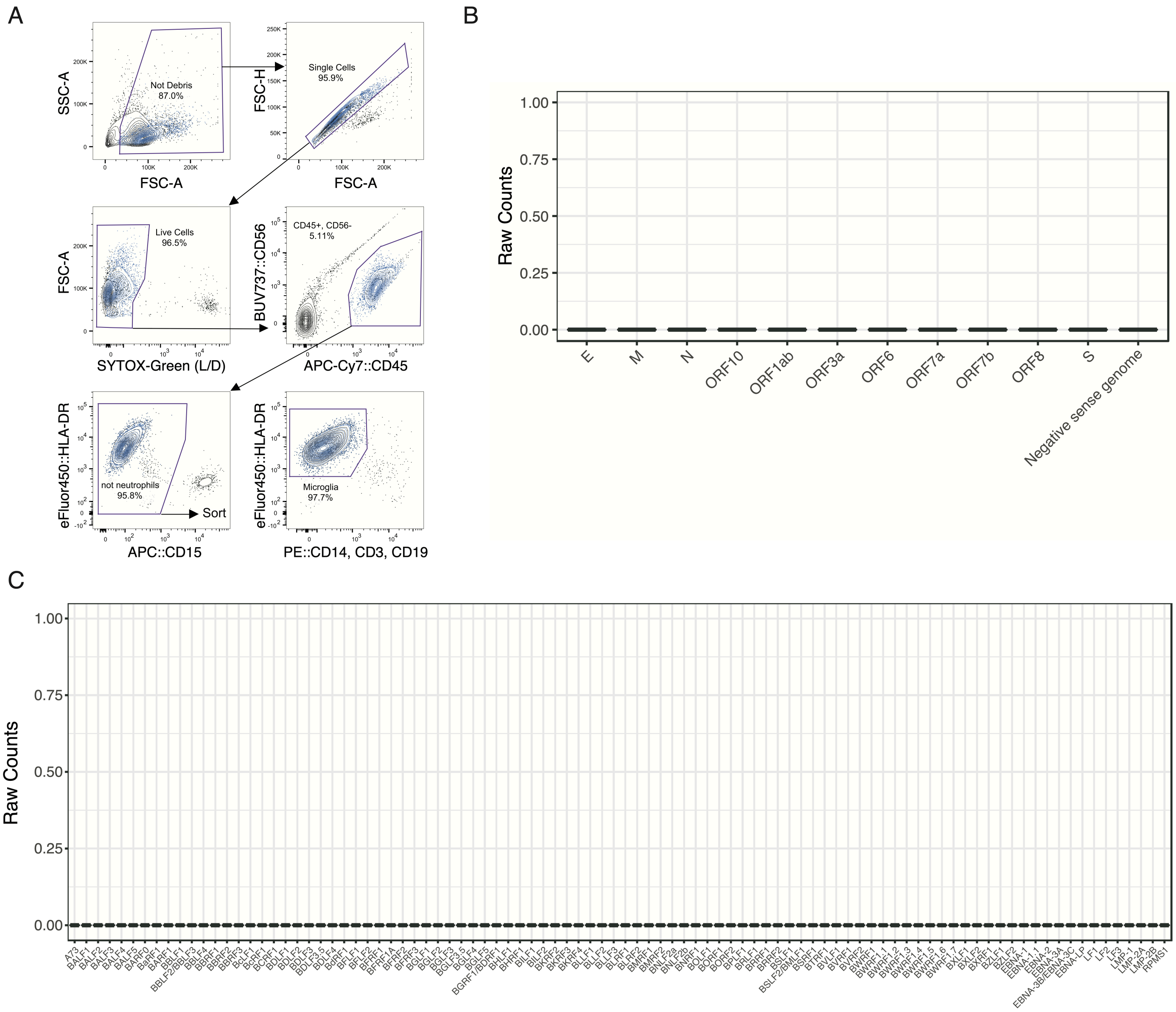

Supplemental Figure S1. Related to figure 1**. (A)** Representative flow cytometry sorting scheme for isolating neuro-immune cells from human postmortem brain tissue from a frontal lobe sample. **(B)** Boxplots of raw gene counts for all genes in the SARS-CoV-2 (NC045512.2) genome from human neuro-immune cell scRNA-seq. Zero counts are detected for all genes. **(C)** Boxplots of raw gene counts for all genes in the EBV1 genome (NC007605.1) from human neuro-immune cell scRNA-seq. Zero counts are detected for all genes.

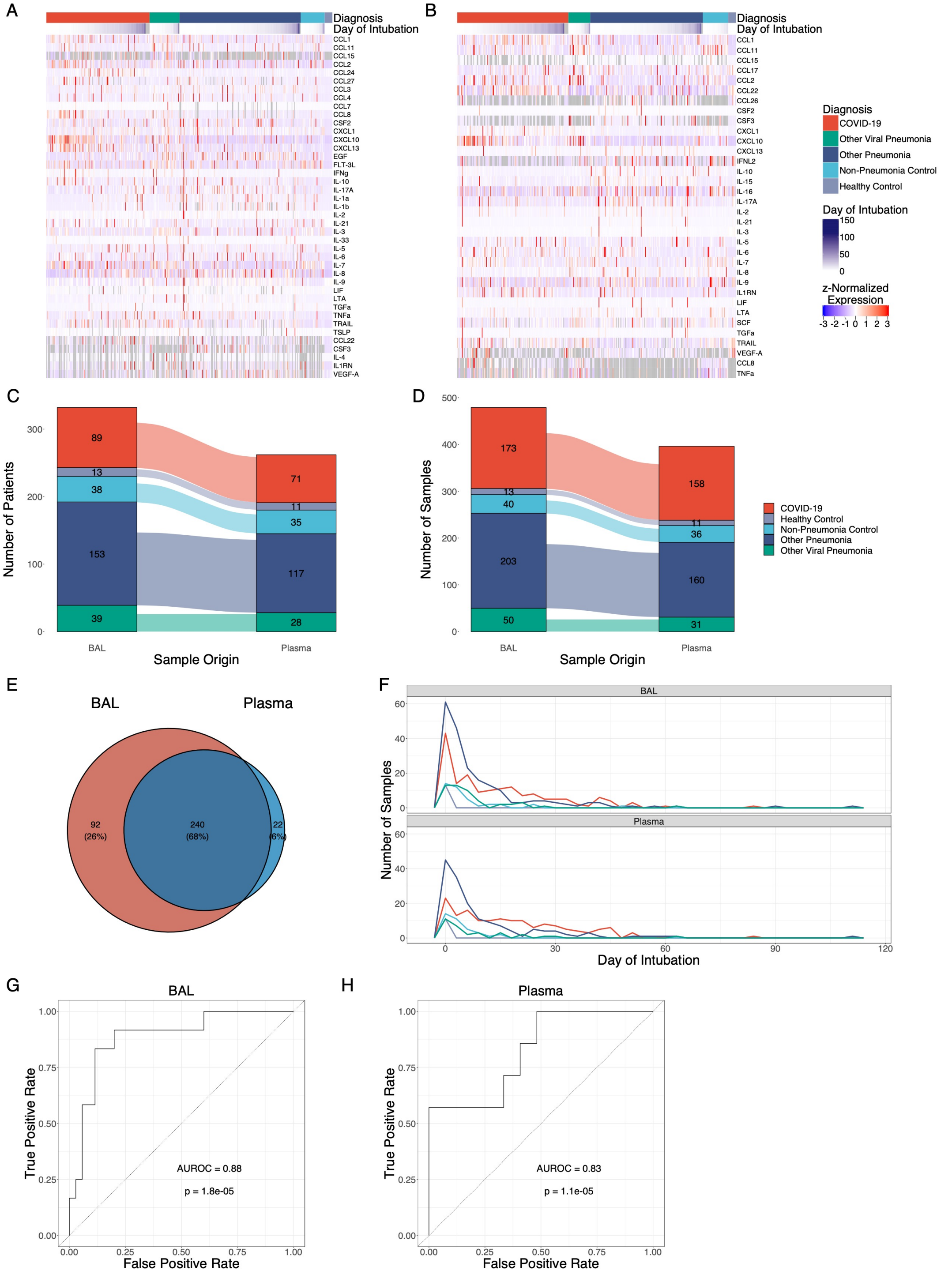

Supplemental Figure S2. Related to figure 2**. (A)** Heatmap of 41 cytokines showing significant variability by diagnosis (q < 0.05, Kruskal–Wallis) from 479 BAL samples collected throughout the entire ICU stay from 332 patients. **(B)** Heatmap of 31 cytokines showing significant variability by diagnosis (q < 0.05, Kruskal–Wallis) from 396 plasma samples collected throughout the entire ICU stay from 262 patients. **(C,D)** Alluvial plot of total numbers of patients included in the BAL fluid (c) and plasma (d) datasets. **(E)** Venn diagram of patients with BAL and plasma samples. **(F)** Frequency polygon plot of BAL and plasma sampling distributions throughout patient stay by group. **(G)** AUROC of prediction of a diagnosis of COVID-19 as compared to all other diagnoses from CXCL10 measurement on initial BAL using a logistic-regression-based machine learning model. **(H)** AUROC of prediction of a diagnosis of COVID-19 as compared to all other diagnoses from CXCL10 measurement on initial plasma draw using a logistic-regression-based machine learning model.

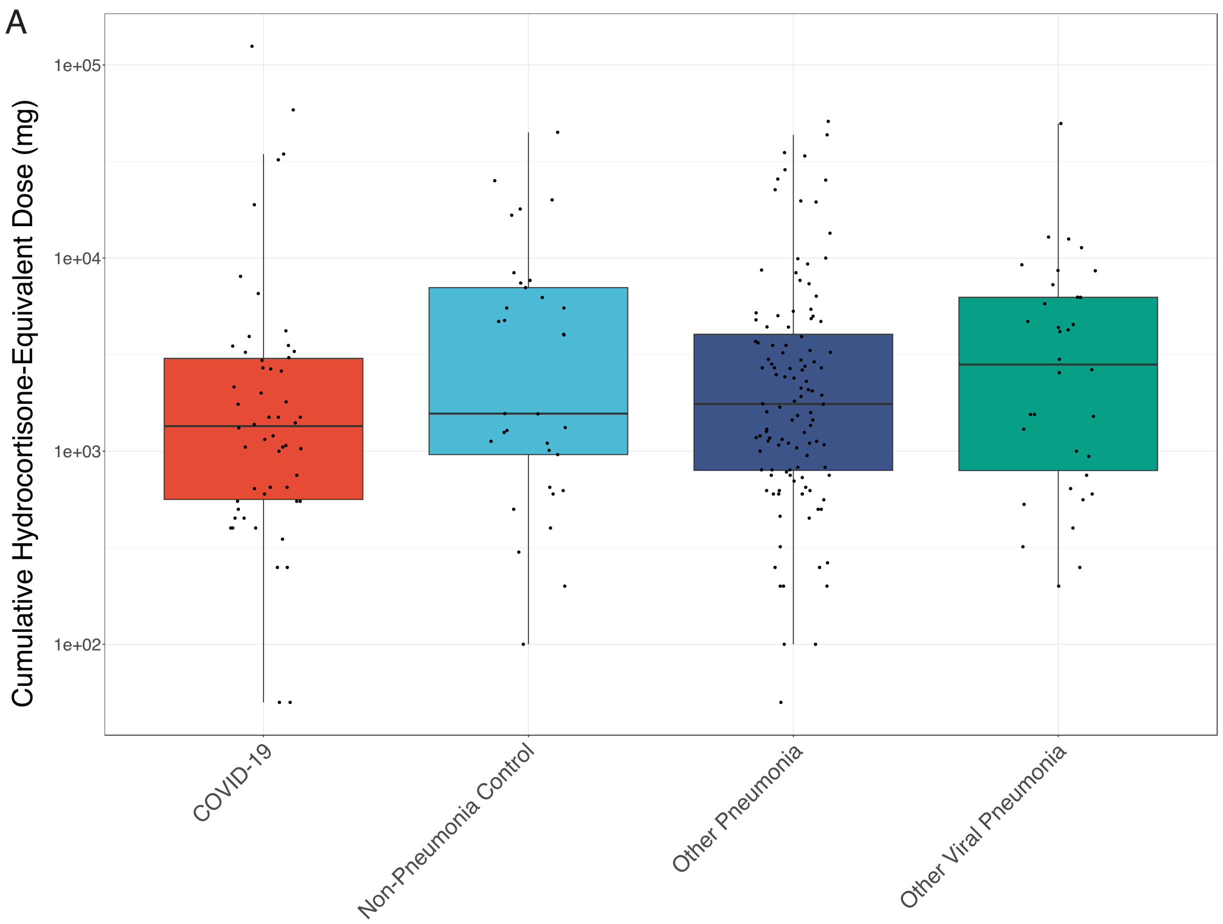

Supplemental Figure S3. Cumulative equivalent steroid dosing by cohort. Cumulative hydrocortisone-equivalent corticosteroid doses were calculated over the course of each patient's ICU stay as previously defined in (1). Patients not receiving a single dose of corticosteroids throughout their ICU stay were discarded for this analysis. No significant differences were observed between cohorts of critically ill patients.

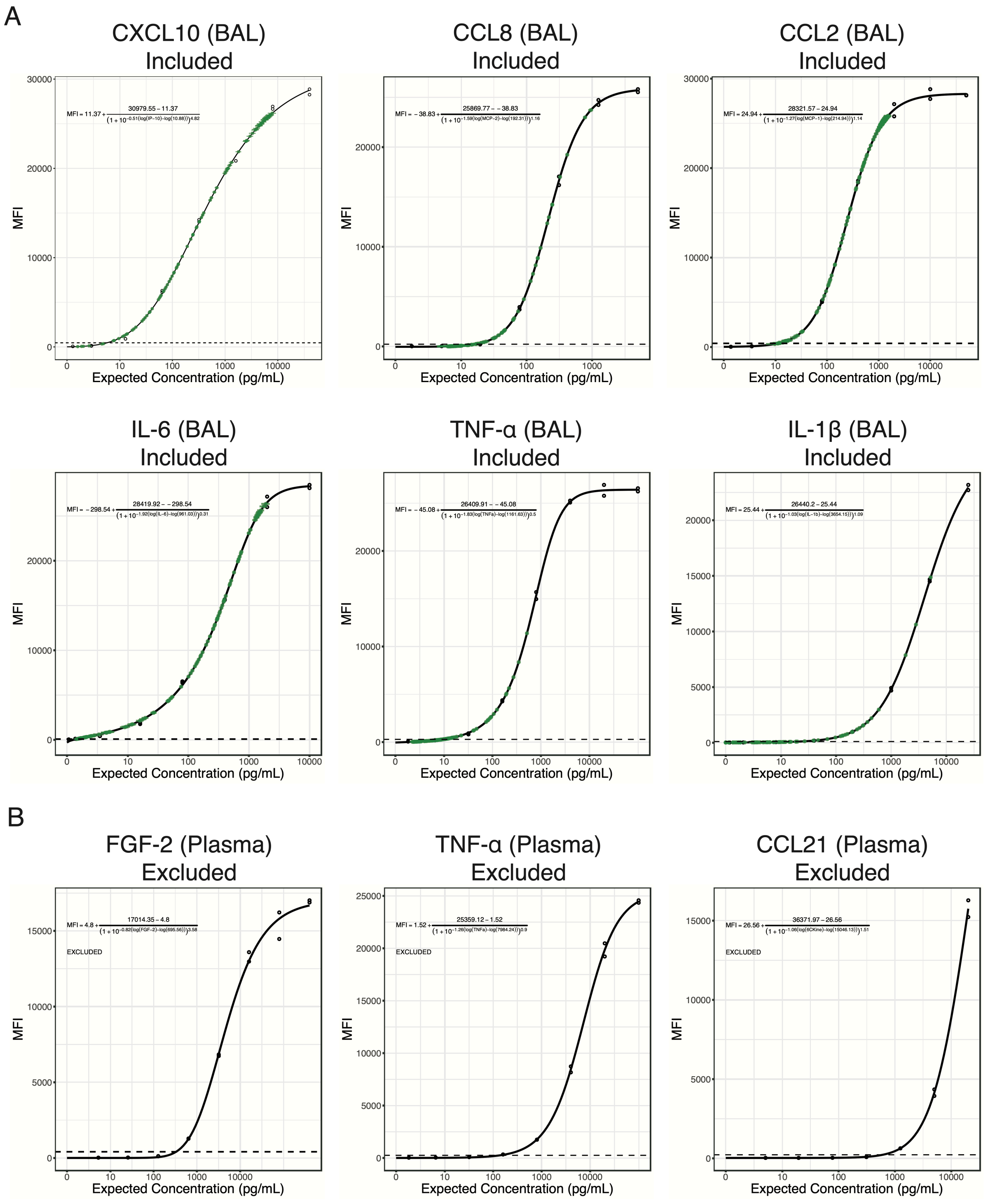

Supplemental Figure S4. Representative standard curve fitting plots for cytokine data exclusion. (A) Representative standard curves and fitted cytokine expression values for analytes included in this study. From left to right, top to bottom: CXCL10 (IP-10), CCL8 (MCP-2), CCL2 (MCP-1) IL-6, TNF-α, IL-1b. **(B)** Representative standard curves for analytes excluded for poor sensitivity in this study. From left to right: FGF-2, TNF-α, and CCL21. For all plots, empty points represent standards of known concentration used for curve-fitting. Black curves and associated formulae represent the function fitted to these points. Green points represent experimental values, and green whiskers are the standard error of prediction. Dotted lines are the upper limit of the 95% confidence interval of lower asymptote prediction.

Supplemental Tables

	COVID-19 (N = 5)	Control (N = 3)	Total (N = 8)
**Age (years)**			
Minimum	35	44	35
Median (IQR)	61.0 (61.0, 72.0)	51.0 (47.5, 63.5)	61.0 (49.2, 73.0)
Mean (SD)	61.4 ± 16.5	57.0 ± 16.8	59.8 ± 15.5
Maximum	78	76	78
**Sex**			
Female	0 (0.0%)	1 (33.3%)	1 (12.5%)
Male	5 (100.0%)	2 (66.7%)	7 (87.5%)
**Race**			
Asian	1 (20.0%)	0 (0.0%)	1 (12.5%)
Black or African-American	1 (20.0%)	1 (33.3%)	2 (25.0%)
Other	0 (0.0%)	1 (33.3%)	1 (12.5%)
White	3 (60.0%)	1 (33.3%)	4 (50.0%)
**Ethnicity**			
Hispanic or Latino	1 (20.0%)	0 (0.0%)	1 (12.5%)
Not Hispanic or Latino	4 (80.0%)	3 (100.0%)	7 (87.5%)
**SOFA**			
Minimum	12	10	10
Median (IQR)	15.0 (13.5, 16.5)	14.5 (12.2, 16.8)	15.0 (12.5, 17.5)
Mean (SD)	15.0 ± 2.6	14.5 ± 6.4	14.8 ± 3.5
Maximum	18	19	19
Unknown/Missing	1 (20.0%)	1 (33.3%)	2 (25.0%)
**Discharge Status**			
Deceased	5 (100.0%)	3 (100.0%)	8 (100.0%)
**Duration of Intubation (days)**			
Minimum	0.8	0.9	0.8
Median (IQR)	7.0 (1.2, 18.0)	1.7 (1.3, 8.8)	4.3 (1.1, 16.5)
Mean (SD)	9.3 ± 9.0	6.2 ± 8.5	8.1 ± 8.3
Maximum	19.4	16	19.4
**Length of ICU Stay (days)**			
Minimum	0.8	0	0
Median (IQR)	7.9 (1.2, 15.4)	1.7 (0.8, 8.8)	4.8 (1.1, 15.6)
Mean (SD)	8.3 ± 7.4	5.9 ± 8.8	7.4 ± 7.4
Maximum	16	16	16
**BMI (kg/m2)**			
Minimum	23.8	20.7	20.7
Median (IQR)	26.4 (24.6, 29.4)	21.3 (21.0, 28.1)	24.9 (22.6, 30.8)
Mean (SD)	27.6 ± 4.4	25.7 ± 8.1	26.8 ± 5.7
Maximum	33.6470588235294	35	35
Unknown/Missing	1 (20.0%)	0 (0.0%)	1 (12.5%)
**Smoking Status**			
Current Smoker	1 (20.0%)	0 (0.0%)	1 (12.5%)
Never Smoker	4 (80.0%)	2 (66.7%)	6 (75.0%)
Past Smoker	0 (0.0%)	1 (33.3%)	1 (12.5%)
**Postmortem Interval (hours)**			
Minimum	15.2	16.7	15.2
Median (IQR)	38.4 (18.9, 45.5)	24.3 (20.5, 36.4)	31.4 (18.3, 46.2)
Mean (SD)	35.1 ± 17.9	29.8 ± 16.6	33.1 ± 16.4
Maximum	57.5	48.5	57.5
**Immunosuppressed**			
Immunosuppressed	2 (40.0%)	0 (0.0%)	2 (25.0%)
Not Immunosuppressed	3 (60.0%)	3 (100.0%)	6 (75.0%)
**Cancer**			
Diagnosed	0 (0.0%)	1 (33.3%)	1 (12.5%)
Not Diagnosed	5 (100.0%)	2 (66.7%)	7 (87.5%)
**Cardiac Arrest**			
Cardiac Arrest with ROSC	2 (40.0%)	2 (66.7%)	4 (50.0%)
No Cardiac Arrest with ROSC	3 (60.0%)	1 (33.3%)	4 (50.0%)
**Cognitive Impairment**			
Diagnosed	1 (20.0%)	1 (33.3%)	2 (25.0%)
Not Diagnosed	4 (80.0%)	2 (66.7%)	6 (75.0%)
**Diabetes**			
Diagnosed	1 (20.0%)	0 (0.0%)	1 (12.5%)
Not Diagnosed	4 (80.0%)	3 (100.0%)	7 (87.5%)
**Renal Disease**			
Diagnosed	3 (60.0%)	1 (33.3%)	4 (50.0%)
Not Diagnosed	2 (40.0%)	2 (66.7%)	4 (50.0%)
**Substance Use Disorder**			
Not Diagnosed	5 (100.0%)	3 (100.0%)	8 (100.0%)
**Venous Thromboembolism**			
Diagnosed	0 (0.0%)	1 (33.3%)	1 (12.5%)

Supplemental Table S1. Demographics of the postmortem brain scRNA-seq cohort. Frontal lobe samples were collected in the course of autopsy at Northwestern Memorial Hospital.

	COVID-19 (N = 5)	Control (N = 3)	Total (N = 8)
Not Diagnosed	5 (100.0%)	2 (66.7%)	7 (87.5%)
**Convalescent Plasma**			
Received Treatment	1 (20.0%)	0 (0.0%)	1 (12.5%)
Did Not Receive Treatment	4 (80.0%)	3 (100.0%)	7 (87.5%)
**Dexamethasone**			
Received Treatment	2 (40.0%)	0 (0.0%)	2 (25.0%)
Did Not Receive Treatment	3 (60.0%)	3 (100.0%)	6 (75.0%)
**IL-6 Blockers**			
Received Treatment	1 (20.0%)	0 (0.0%)	1 (12.5%)
Did Not Receive Treatment	4 (80.0%)	3 (100.0%)	7 (87.5%)
**Remdesivir**			
Received Treatment	1 (20.0%)	0 (0.0%)	1 (12.5%)
Did Not Receive Treatment	4 (80.0%)	3 (100.0%)	7 (87.5%)
**CRP (mg/L)**			
Minimum	137.3	Inf	137.3
Median (IQR)	283.6 (210.5, 356.8)	NA (NA, NA)	283.6 (210.5, 356.8)
Mean (SD)	283.6 ± 207.0	NaN ± NA	283.6 ± 207.0
Maximum	430	-Inf	430
Unknown/Missing	3 (60.0%)	3 (100.0%)	6 (75.0%)
**D-Dimer (ng/mL)**			
Minimum	1135	6226	1135
Median (IQR)	3,078.5 (2,106.8, 4,050.2)	6,226.0 (6,226.0, 6,226.0)	5,022.0 (3,078.5, 5,624.0)
Mean (SD)	3,078.5 ± 2,748.5	6,226.0 ± NA	4,127.7 ± 2,660.7
Maximum	5022	6226	6226
Unknown/Missing	3 (60.0%)	2 (66.7%)	5 (62.5%)

Supplemental Table S1. Demographics of the postmortem brain scRNA-seq cohort. Frontal lobe samples were collected in the course of autopsy at Northwestern Memorial Hospital.

	COVID-19 (N = 93)	Healthy Control (N = 13)	Non-Pneumonia Control (N = 45)	Other Pneumonia (N = 162)	Other Viral Pneumonia (N = 41)	Total (N = 354)
**Age (years)**						
Minimum	21	21	19	22	34	19
Median (IQR)	59.0 (46.0, 68.2)	25.0 (23.0, 33.0)	62.0 (49.0, 72.0)	66.0 (51.2, 73.0)	59.0 (52.0, 69.0)	62.0 (48.0, 72.0)
Mean (SD)	57.1 ± 14.3	27.8 ± 6.7	59.2 ± 17.6	61.8 ± 16.3	60.3 ± 13.1	58.9 ± 16.6
Maximum	86	40	90	99	88	99
Unknown/Missing	5 (5.4%)	0 (0.0%)	0 (0.0%)	0 (0.0%)	0 (0.0%)	5 (1.4%)
**Sex**						
Female	31 (33.3%)	7 (53.8%)	20 (44.4%)	63 (38.9%)	18 (43.9%)	139 (39.3%)
Male	60 (64.5%)	6 (46.2%)	25 (55.6%)	99 (61.1%)	23 (56.1%)	213 (60.2%)
Unknown or Not Reported	2 (2.2%)	0 (0.0%)	0 (0.0%)	0 (0.0%)	0 (0.0%)	2 (0.6%)
**Race**						
White	36 (38.7%)	8 (61.5%)	28 (62.2%)	109 (67.3%)	26 (63.4%)	207 (58.5%)
Black/African American	19 (20.4%)	1 (7.7%)	8 (17.8%)	34 (21.0%)	8 (19.5%)	70 (19.8%)
Unknown or Not Reported	37 (39.8%)	0 (0.0%)	6 (13.3%)	13 (8.0%)	5 (12.2%)	61 (17.2%)
Asian	<=5	<=5	<=5	<=5	<=5	14 (4.0%)
American Indian/Alaska Native	<=5	<=5	<=5	<=5	<=5	<=5
**Ethnicity**						
Hispanic or Latino	40 (43.0%)	2 (15.4%)	7 (15.6%)	13 (8.0%)	8 (19.5%)	70 (19.8%)
Not Hispanic or Latino	39 (41.9%)	11 (84.6%)	35 (77.8%)	137 (84.6%)	30 (73.2%)	252 (71.2%)
Unknown or Not Reported	14 (15.1%)	0 (0.0%)	3 (6.7%)	12 (7.4%)	3 (7.3%)	32 (9.0%)
**SOFA**						
Minimum	4	Inf	3	4	2	2
Median (IQR)	9.0 (7.6, 11.2)	NA (NA, NA)	10.8 (8.2, 13.2)	11.0 (9.0, 13.9)	9.5 (8.2, 12.5)	10.1 (8.1, 13.0)
Mean (SD)	9.7 ± 2.8	NaN ± NA	10.8 ± 3.6	11.4 ± 3.6	10.2 ± 3.0	10.7 ± 3.4
Maximum	18.5	-Inf	20.3	21.2	15.3	21.2
Unknown/Missing	2 (2.2%)	13 (100.0%)	5 (11.1%)	15 (9.3%)	7 (17.1%)	42 (11.9%)
**APS**						
Minimum	39.2	Inf	35.2	23	23.8	23
Median (IQR)	72.3 (61.5, 86.4)	NA (NA, NA)	66.6 (51.0, 78.6)	66.2 (53.6, 80.4)	63.3 (56.4, 77.3)	68.4 (56.0, 81.1)
Mean (SD)	73.6 ± 17.7	NaN ± NA	67.5 ± 19.1	68.2 ± 21.3	66.0 ± 19.0	69.3 ± 20.0
Maximum	108.6	-Inf	109.8	161	105	161
Unknown/Missing	2 (2.2%)	13 (100.0%)	0 (0.0%)	0 (0.0%)	0 (0.0%)	15 (4.2%)
**Discharge Status**						
Deceased	26 (28.6%)	0 (NaN%)	15 (33.3%)	59 (36.4%)	14 (34.1%)	114 (33.6%)
Home	36 (39.6%)	0 (NaN%)	20 (44.4%)	36 (22.2%)	10 (24.4%)	102 (30.1%)
Inpatient Facility	17 (18.7%)	0 (NaN%)	7 (15.6%)	48 (29.6%)	14 (34.1%)	86 (25.4%)
LTAC	12 (13.2%)	0 (NaN%)	3 (6.7%)	18 (11.1%)	3 (7.3%)	36 (10.6%)
Other	0 (0.0%)	0 (NaN%)	0 (0.0%)	1 (0.6%)	0 (0.0%)	1 (0.3%)
Unknown/Missing	2 (2.2%)	13 (100.0%)	0 (0.0%)	0 (0.0%)	0 (0.0%)	15 (4.2%)
**Duration of Intubation (days)**						
Minimum	0	0	0	0	1	0
Median (IQR)	20.0 (10.0, 32.0)	0.0 (0.0, 0.0)	4.0 (2.0, 9.0)	8.0 (3.0, 17.0)	9.0 (3.0, 14.0)	9.0 (3.0, 19.0)
Mean (SD)	23.9 ± 21.6	0.0 ± 0.0	8.4 ± 11.6	11.7 ± 11.6	10.4 ± 8.9	13.9 ± 15.8
Maximum	153	0	58	68	34	153
**Length of ICU Stay (days)**						
Minimum	3	0	1	1	2	0
Median (IQR)	22.0 (13.5, 35.0)	0.0 (0.0, 0.0)	7.0 (4.0, 12.0)	10.0 (5.0, 19.0)	11.0 (7.0, 20.0)	12.0 (5.0, 22.0)
Mean (SD)	26.6 ± 21.2	0.0 ± 0.0	11.3 ± 13.9	14.7 ± 14.2	13.4 ± 8.9	16.6 ± 16.8
Maximum	153	0	80	86	34	153
Unknown/Missing	2 (2.2%)	0 (0.0%)	0 (0.0%)	0 (0.0%)	0 (0.0%)	2 (0.6%)
**BMI (kg/m2)**						
Minimum	19.22	18.4	16.56	12.46	17.34	12.46
Median (IQR)	31.8 (27.9, 38.9)	24.1 (22.3, 26.0)	27.2 (24.5, 33.5)	26.6 (21.9, 31.6)	26.8 (23.2, 31.3)	28.1 (23.9, 33.8)
Mean (SD)	34.1 ± 9.1	24.8 ± 4.2	30.7 ± 9.9	27.5 ± 8.3	29.0 ± 9.3	29.7 ± 9.1
Maximum	81.81	35.1	70.6	70.25	64.18	81.81
Unknown/Missing	3 (3.2%)	0 (0.0%)	1 (2.2%)	1 (0.6%)	0 (0.0%)	5 (1.4%)
**Smoking Status**						
Current Smoker	0 (0.0%)	0 (0.0%)	6 (13.3%)	21 (13.0%)	5 (12.2%)	32 (9.0%)
Never Smoker	36 (38.7%)	13 (100.0%)	25 (55.6%)	65 (40.1%)	20 (48.8%)	159 (44.9%)
Past Smoker	13 (14.0%)	0 (0.0%)	10 (22.2%)	59 (36.4%)	15 (36.6%)	97 (27.4%)
Unknown or Not Reported	44 (47.3%)	0 (0.0%)	4 (8.9%)	17 (10.5%)	1 (2.4%)	66 (18.6%)
**Immunocompromised**						
Immunocompromised	9 (9.9%)	0 (0.0%)	10 (22.2%)	49 (30.2%)	18 (43.9%)	86 (24.4%)
Not Immunocompromised	82 (90.1%)	13 (100.0%)	35 (77.8%)	113 (69.8%)	23 (56.1%)	266 (75.6%)
Unknown/Missing	2 (2.2%)	0 (0.0%)	0 (0.0%)	0 (0.0%)	0 (0.0%)	2 (0.6%)
**AIDS/HIV**						
Diagnosed	1 (1.1%)	0 (0.0%)	0 (0.0%)	2 (1.2%)	3 (7.3%)	6 (1.7%)
Not Diagnosed	90 (98.9%)	13 (100.0%)	45 (100.0%)	160 (98.8%)	38 (92.7%)	346 (98.3%)
Unknown/Missing	2 (2.2%)	0 (0.0%)	0 (0.0%)	0 (0.0%)	0 (0.0%)	2 (0.6%)
**Cancer**						
Diagnosed	14 (15.4%)	0 (0.0%)	15 (33.3%)	58 (35.8%)	21 (51.2%)	108 (30.7%)

Supplemental Table S2. Demographics of the SCRIPT cytokines cohort.

	COVID-19 (N = 93)	Healthy Control (N = 13)	Non-Pneumonia Control (N = 45)	Other Pneumonia (N = 162)	Other Viral Pneumonia (N = 41)	Total (N = 354)
Not Diagnosed	77 (84.6%)	13 (100.0%)	30 (66.7%)	104 (64.2%)	20 (48.8%)	244 (69.3%)
Unknown/Missing	2 (2.2%)	0 (0.0%)	0 (0.0%)	0 (0.0%)	0 (0.0%)	2 (0.6%)
**Cerebrovascular Disease**						
Diagnosed	9 (9.9%)	0 (0.0%)	9 (20.0%)	39 (24.1%)	10 (24.4%)	67 (19.0%)
Not Diagnosed	82 (90.1%)	13 (100.0%)	36 (80.0%)	123 (75.9%)	31 (75.6%)	285 (81.0%)
Unknown/Missing	2 (2.2%)	0 (0.0%)	0 (0.0%)	0 (0.0%)	0 (0.0%)	2 (0.6%)
**Chronic Pulmonary Disease**						
Diagnosed	21 (23.1%)	0 (0.0%)	16 (35.6%)	68 (42.0%)	17 (41.5%)	122 (34.7%)
Not Diagnosed	70 (76.9%)	13 (100.0%)	29 (64.4%)	94 (58.0%)	24 (58.5%)	230 (65.3%)
Unknown/Missing	2 (2.2%)	0 (0.0%)	0 (0.0%)	0 (0.0%)	0 (0.0%)	2 (0.6%)
**Congestive Heart Failure**						
Diagnosed	14 (15.4%)	0 (0.0%)	19 (42.2%)	58 (35.8%)	7 (17.1%)	98 (27.8%)
Not Diagnosed	77 (84.6%)	13 (100.0%)	26 (57.8%)	104 (64.2%)	34 (82.9%)	254 (72.2%)
Unknown/Missing	2 (2.2%)	0 (0.0%)	0 (0.0%)	0 (0.0%)	0 (0.0%)	2 (0.6%)
**Dementia**						
Diagnosed	3 (3.3%)	0 (0.0%)	4 (8.9%)	4 (2.5%)	3 (7.3%)	14 (4.0%)
Not Diagnosed	88 (96.7%)	13 (100.0%)	41 (91.1%)	158 (97.5%)	38 (92.7%)	338 (96.0%)
Unknown/Missing	2 (2.2%)	0 (0.0%)	0 (0.0%)	0 (0.0%)	0 (0.0%)	2 (0.6%)
**Diabetes**						
Diagnosed	41 (45.1%)	0 (0.0%)	14 (31.1%)	44 (27.2%)	21 (51.2%)	120 (34.1%)
Not Diagnosed	50 (54.9%)	13 (100.0%)	31 (68.9%)	118 (72.8%)	20 (48.8%)	232 (65.9%)
Unknown/Missing	2 (2.2%)	0 (0.0%)	0 (0.0%)	0 (0.0%)	0 (0.0%)	2 (0.6%)
**Hemiplagia or Paraplegia**						
Diagnosed	2 (2.2%)	0 (0.0%)	5 (11.1%)	17 (10.5%)	3 (7.3%)	27 (7.7%)
Not Diagnosed	89 (97.8%)	13 (100.0%)	40 (88.9%)	145 (89.5%)	38 (92.7%)	325 (92.3%)
Unknown/Missing	2 (2.2%)	0 (0.0%)	0 (0.0%)	0 (0.0%)	0 (0.0%)	2 (0.6%)
**Myocardial Infarction**						
Diagnosed	2 (2.2%)	0 (0.0%)	8 (17.8%)	15 (9.3%)	2 (4.9%)	27 (7.7%)
Not Diagnosed	89 (97.8%)	13 (100.0%)	37 (82.2%)	147 (90.7%)	39 (95.1%)	325 (92.3%)
Unknown/Missing	2 (2.2%)	0 (0.0%)	0 (0.0%)	0 (0.0%)	0 (0.0%)	2 (0.6%)
**Peripheral Vascular Disease**						
Diagnosed	12 (13.2%)	0 (0.0%)	10 (22.2%)	44 (27.2%)	8 (19.5%)	74 (21.0%)
Not Diagnosed	79 (86.8%)	13 (100.0%)	35 (77.8%)	118 (72.8%)	33 (80.5%)	278 (79.0%)
Unknown/Missing	2 (2.2%)	0 (0.0%)	0 (0.0%)	0 (0.0%)	0 (0.0%)	2 (0.6%)
**Renal Disease**						
Diagnosed	13 (14.3%)	0 (0.0%)	20 (44.4%)	50 (30.9%)	12 (29.3%)	95 (27.0%)
Not Diagnosed	78 (85.7%)	13 (100.0%)	25 (55.6%)	112 (69.1%)	29 (70.7%)	257 (73.0%)
Unknown/Missing	2 (2.2%)	0 (0.0%)	0 (0.0%)	0 (0.0%)	0 (0.0%)	2 (0.6%)
**Rheumatic Disease**						
Diagnosed	4 (4.4%)	0 (0.0%)	5 (11.1%)	15 (9.3%)	4 (9.8%)	28 (8.0%)
Not Diagnosed	87 (95.6%)	13 (100.0%)	40 (88.9%)	147 (90.7%)	37 (90.2%)	324 (92.0%)
Unknown/Missing	2 (2.2%)	0 (0.0%)	0 (0.0%)	0 (0.0%)	0 (0.0%)	2 (0.6%)
**Charlson Comorbidity Index**						
Minimum	1	Inf	1	1	1	1
Median (IQR)	3 (1.0, 5.0)	NA (NA, NA)	6 (4.0, 9.0)	5 (3.0, 8.0)	5 (3.0, 8.0)	5.0 (2.0, 8.0)
Mean (SD)	3.9 ± 3.7	NaN ± NA	6.3 ± 3.4	5.7 ± 3.5	6.1 ± 4.0	5.4 ± 3.7
Maximum	18	-Inf	14	17	21	21
Unknown/Missing	36 (38.7%)	13 (100.0%)	8 (17.8%)	25 (15.4%)	4 (9.8%)	86 (24.3%)
**Steroids**						
Treated	54 (58.1%)	0 (0.0%)	33 (73.3%)	116 (71.6%)	34 (82.9%)	237 (66.9%)
Untreated	39 (41.9%)	13 (100.0%)	12 (26.7%)	46 (28.4%)	7 (17.1%)	117 (33.1%)
**CRP (mg/L)**						
Minimum	0	Inf	0	0	16.8	0
Median (IQR)	17.1 (10.7, 28.8)	NA (NA, NA)	2.8 (1.2, 8.2)	8.6 (2.8, 15.3)	29.5 (23.1, 32.8)	15.3 (6.0, 27.1)
Mean (SD)	22.9 ± 25.7	NaN ± NA	8.8 ± 15.2	10.3 ± 9.4	27.4 ± 9.8	19.4 ± 23.0
Maximum	197.3	-Inf	48.3	32.2	36	197.3
Unknown/Missing	4 (4.3%)	13 (100.0%)	36 (80.0%)	136 (84.0%)	38 (92.7%)	227 (64.1%)
**D-Dimer (ng/mL)**						
Minimum	0	Inf	187	0	181	0
Median (IQR)	1,069.0 (512.0, 3,573.0)	NA (NA, NA)	3,507.0 (2,734.2, 4,712.2)	2,237.0 (733.0, 6,360.0)	2,398.0 (876.0, 3,315.0)	1,905.0 (610.0, 4,622.0)
Mean (SD)	2,777.3 ± 3,680.2	NaN ± NA	10,501.4 ± 18,742.0	5,482.0 ± 9,262.9	3,229.5 ± 3,327.0	4,328.7 ± 8,142.1
Maximum	18329	-Inf	55024	48657	11692	55024
Unknown/Missing	4 (4.3%)	13 (100.0%)	31 (68.9%)	101 (62.3%)	24 (58.5%)	173 (48.9%)
**Ferritin (ng/mL)**						
Minimum	66.5	Inf	21.6	33.3	184.9	21.6
Median (IQR)	798.5 (380.6, 1,394.2)	NA (NA, NA)	250.5 (92.4, 290.1)	376.7 (135.9, 1,257.8)	938.1 (599.2, 1,166.8)	677.9 (257.4, 1,361.5)
Mean (SD)	1,671.3 ± 3,383.9	NaN ± NA	298.8 ± 321.8	1,367.2 ± 4,169.4	1,095.3 ± 904.7	1,498.1 ± 3,456.3
Maximum	25815	-Inf	839.5	23599.7	2781.7	25815
Unknown/Missing	21 (22.6%)	13 (100.0%)	40 (88.9%)	131 (80.9%)	35 (85.4%)	240 (67.8%)
**WBC Count, BAL (cells/µL)**						
Minimum	31	Inf	0	3	0	0

Supplemental Table S2. Demographics of the SCRIPT cytokines cohort.

	COVID-19 (N = 93)	Healthy Control (N = 13)	Non-Pneumonia Control (N = 45)	Other Pneumonia (N = 162)	Other Viral Pneumonia (N = 41)	Total (N = 354)
Median (IQR)	207.5 (127.2, 434.0)	NA (NA, NA)	146.5 (33.2, 251.0)	423.5 (193.8, 1,612.2)	781.5 (177.0, 2,147.0)	273.5 (143.8, 863.2)
Mean (SD)	848.3 ± 3,873.2	NaN ± NA	495.5 ± 1,403.1	2,434.8 ± 5,691.9	2,038.4 ± 4,215.8	1,661.5 ± 4,674.0
Maximum	34300	-Inf	7800	43375	24500	43375
Unknown/Missing	11 (11.8%)	13 (100.0%)	3 (6.7%)	30 (18.5%)	5 (12.2%)	62 (17.5%)
**Neutrophils, BAL (%)**						
Minimum	2	Inf	0	0	0	0
Median (IQR)	41.5 (22.5, 69.0)	NA (NA, NA)	26.0 (6.0, 56.8)	82.0 (63.8, 91.2)	84.0 (57.5, 90.0)	69.0 (32.0, 87.0)
Mean (SD)	45.4 ± 27.2	NaN ± NA	34.4 ± 30.3	72.9 ± 25.0	66.8 ± 32.8	59.7 ± 31.2
Maximum	95	-Inf	97	100	96	100
Unknown/Missing	11 (11.8%)	13 (100.0%)	3 (6.7%)	14 (8.6%)	3 (7.3%)	44 (12.4%)
**Lymphocytes, BAL (%)**						
Minimum	1	Inf	1	1	1	1
Median (IQR)	14.0 (6.5, 29.5)	NA (NA, NA)	5.0 (3.0, 8.8)	3.0 (1.0, 6.0)	2.0 (1.0, 5.2)	5.0 (2.0, 12.0)
Mean (SD)	20.5 ± 18.7	NaN ± NA	8.8 ± 15.1	5.7 ± 8.2	5.8 ± 10.5	10.7 ± 14.9
Maximum	85	-Inf	88	51	55	88
Unknown/Missing	14 (15.1%)	13 (100.0%)	11 (24.4%)	48 (29.6%)	9 (22.0%)	95 (26.8%)
**WBC Count, Blood (10e3 cells/µL)**						
Minimum	3.4	Inf	4.8	0.1	5.2	0.1
Median (IQR)	10.2 (7.2, 14.2)	NA (NA, NA)	9.2 (6.9, 11.5)	11.9 (9.1, 15.8)	8.7 (7.7, 10.2)	10.4 (7.6, 14.5)
Mean (SD)	12.5 ± 10.8	NaN ± NA	9.9 ± 4.8	13.9 ± 8.7	9.2 ± 3.8	12.6 ± 9.9
Maximum	98.6	-Inf	20.4	43.6	14.4	98.6
Unknown/Missing	4 (4.3%)	13 (100.0%)	36 (80.0%)	128 (79.0%)	37 (90.2%)	218 (61.6%)
**Absolute Neutrophils, Blood (10e3 cells/µL)**						
Minimum	2.3	Inf	0.6	0.1	0	0
Median (IQR)	8.1 (5.4, 10.7)	NA (NA, NA)	11.2 (5.7, 17.9)	10.0 (5.8, 15.6)	7.4 (4.9, 13.0)	9.0 (5.4, 14.1)
Mean (SD)	8.9 ± 4.6	NaN ± NA	12.3 ± 8.5	11.7 ± 8.5	9.3 ± 7.3	10.7 ± 7.5
Maximum	25.4	-Inf	32.3	48.3	27.1	48.3
Unknown/Missing	9 (9.7%)	13 (100.0%)	11 (24.4%)	25 (15.4%)	8 (19.5%)	66 (18.6%)
**Absolute Lymphocytes, Blood (10e3 cells/µL)**						
Minimum	0	Inf	0	0	0	0
Median (IQR)	1.0 (0.6, 1.4)	NA (NA, NA)	0.6 (0.4, 1.4)	0.9 (0.5, 1.8)	0.8 (0.3, 1.6)	0.9 (0.5, 1.6)
Mean (SD)	2.3 ± 10.1	NaN ± NA	1.4 ± 2.1	1.6 ± 2.3	1.1 ± 0.9	1.7 ± 5.7
Maximum	93.7	-Inf	11.2	18.2	3.8	93.7
Unknown/Missing	9 (9.7%)	13 (100.0%)	11 (24.4%)	24 (14.8%)	8 (19.5%)	65 (18.4%)

Supplemental Table S2. Demographics of the SCRIPT cytokines cohort.

Laser	Filter	Dye	Antigen	Clone	Dilution	Catalog Number	RR ID
305	750/50	BUV737	CD56	NCAM16.2	1:10	BD 612766	AB_2813880
405	450/50	eFluor450	HLA-DR	L243	1:20	Thermo-Fisher 48-9952-42	AB_1603291
552	575/25	PE	CD14	MΦP9	1:10	BD 562691	AB_2737725
552	575/25	PE	CD3	SK7	1:20	Thermo-Fisher 12-0036-42	AB_10805512
552	575/25	PE	CD19	J3-119	1:10	Beckman Coulter IM1285U	AB_10640419
488	530/30	SYTOX Green			1:500-1:1000	Thermo-Fisher S7020	
640	670/39	APC	CD15	W6D3	1:10	BioLegend 323008	AB_756014
640	780/60	APC-Cy7	CD45	HI30	1:10	CST 77322	AB_2799894

Supplemental Table S3. List of reagents used for FACS sorting of human neuroimmune cells.

References

1. Meikle AW, Tyler FH. Potency and duration of action of glucocorticoids: Effects of hydrocortisone, prednisone and dexamethasone on human pituitary-adrenal function. *The American Journal of Medicine*. 1977;63(2):200–207.

Full Name	ORCID	Affiliation 1	Affiliation 2	Affiliation 3
Hiam Abdala-Valencia	0000-0003-3384-5299	Division of Pulmonary and Critical Care Medicine		
Michael J. Alexander	0000-0001-6271-3426	Division of Pulmonary and Critical Care Medicine		
Arghavan Alisoltanidehkordi	0000-0003-3638-2218	Department of Microbiology-Immunology		
Jason M. Arnold	0000-0001-8687-0063	Department of Medicine		
Joseph Isaac Bailey	0000-0002-4225-3108	Division of Pulmonary and Critical Care Medicine		
Elizabeth T. Bartom	0000-0002-5618-2582	Department of Biochemistry and Molecular Genetics		
Ankit Bharat	0000-0002-1248-0457	Division of Thoracic Surgery	Division of Pulmonary and Critical Care Medicine	
Thomas Bolig	0000-0002-8044-4108	Division of Pulmonary and Critical Care Medicine		
Nicole Borkowski	0000-0001-8650-6456	Division of Pulmonary and Critical Care Medicine		
GR Scott Budinger	0000-0002-3114-5208	Division of Pulmonary and Critical Care Medicine		
Navdeep S. Chandel	0000-0001-7208-3886	Division of Pulmonary and Critical Care Medicine		
Rebecca K. Clepp	0000-0002-8197-5103	Division of Pulmonary and Critical Care Medicine		
John Coleman	0000-0003-1246-3814	Division of Pulmonary and Critical Care Medicine		
Michael J. Cuttica	0000-0002-0084-8760	Division of Pulmonary and Critical Care Medicine		
Thaddeus R. Cybulski	0000-0003-0613-7617	Division of Pulmonary and Critical Care Medicine		
Jane E. Dematte	0000-0003-4907-9525	Division of Pulmonary and Critical Care Medicine		
Joseph S. Deters	0000-0002-3522-3824	Division of Pulmonary and Critical Care Medicine		
Estefani Diaz	0000-0003-4146-0276	Robert H. Lurie Comprehensive Cancer Research Center		
Alvaro Donayre	0000-0003-1877-0655	Division of Pulmonary and Critical Care Medicine		
Helen K. Donnelly	0000-0001-8827-8581	Division of Pulmonary and Critical Care Medicine		
Justin A. Fiala	0000-0001-5196-7076	Division of Pulmonary and Critical Care Medicine		
Gaurav T. Gadhvi	0000-0002-4379-2824	Division of Rheumatology		
Catherine A. Gao	0000-0001-5576-3943	Division of Pulmonary and Critical Care Medicine		
Khalilah L. Gates	0000-0002-5965-8136	Division of Pulmonary and Critical Care Medicine		
Samuel W.M. Gatesy	0000-0001-5271-8143	Division of Infectious Disease		
Ritika Giri	0000-0001-8838-0818	Department of Chemical and Biological Engineering		
Pearl D. Go	0000-0003-0533-1864	NU Clinical and Translational Sciences Institute		
Cara J. Gottardi	0000-0003-0912-7617	Division of Pulmonary and Critical Care Medicine		
Rogan A. Grant	0000-0003-0655-0882	Division of Pulmonary and Critical Care Medicine		
Stefan J. Green	0000-0003-2781-359X	Genomics and Microbiome Core Facility, Rush University Medical Center		
Elen Gusman	0000-0002-9415-3168	Division of Pulmonary and Critical Care Medicine		
Estefany R. Guzman	0000-0002-1399-7546	Lurie Cancer Center; Feinberg School of Medicine		

SeungHye Han	0000-0001-5625-6337	Division of Pulmonary and Critical Care Medicine	
Erica Marie Hartmann	0000-0002-0966-2014	Department of Civil & Environmental Engineering	
Alan R. Hauser	0000-0003-4596-7939	Department of Microbiology-Immunology	
Curt M. Horvath	0000-0003-2449-225X	Dept of Molecular Biosciences, Medicine, Microbiology-Immunology	
Mishaal Hukamdad	0000-0002-9508-1688	Division of Pulmonary and Critical Care Medicine	
Judd Hultquist	0000-0001-6424-4280	Division of Infectious Disease	
Sydney M. Hyder	0000-0002-6395-9062	Pulmonary and Critical Care Medicine	
Manu Jain	0000-0003-1534-5629	Division of Pulmonary and Critical Care Medicine	
Emmy Jonasson	0000-0003-4594-5377	Robert H. Lurie Comprehensive Cancer Research Center	
Anthony M. Joudi	0000-0003-1232-3256	Division of Pulmonary and Critical Care Medicine	
Rachel B. Kadar	0000-0002-2583-3637	Critical Care Medicine, Anesthesiology	
Ravi Kalhan	0000-0003-2443-0876	Division of Pulmonary and Critical Care Medicine	
David W. Kamp	0000-0001-5796-1206	Division of Pulmonary and Critical Care Medicine	
Manoj Kandpal	0000-0002-0595-8627	Division of Health and Biomedical Informatics	Division of Organ Transplantation
Mengjia Kang	0000-0002-1679-9473	Division of Pulmonary and Critical Care Medicine	
David A. Kidd	0000-0003-0411-9511	Pulmonary and Critical Care Medicine	
Hermon Kihshen	0000-0002-8750-0010	Division of Pulmonary and Critical Care Medicine	
Zasu M. Klug	0000-0003-3483-396X	Division of Pulmonary and Critical Care Medicine	
Erin A. Korth	0000-003-3853-3219	Division of Pulmonary and Critical Care Medicine	
Jacqueline M. Kruser	0000-0003-3258-1869	Division of Pulmonary and Critical Care Medicine	
Romy Lawrence	0000-0002-6578-8519	Pulmonary and Critical Care Medicine	
Emily M. Leibenguth	0000-0001-7809-7947	Division of Pulmonary and Critical Care Medicine	
Anne R. Levenson	0000-0002-7587-1885	Division of Pulmonary and Critical Care Medicine	
Lindsey D. Gradone	0000-0001-8145-2997	Division of Pulmonary and Critical Care Medicine	
Gabrielle Y. Liu	0000-0003-2049-423	Division of Pulmonary and Critical Care Medicine	
Jon W Lomasney	0000-0002-0438-2427	Department of Pathology	Department of Pharmacology
Theresa A. Lombardo	0000-0002-7468-7541	Division of Pulmonary and Critical Care Medicine	
Ramon Lorenzo-Redondo	0000-0002-5462-9483	Division of Infectious Disease	
Ziyan Lu	0000-0003-2901-3801	Division of Pulmonary and Critical Care Medicine	
Amy Ludwig	0000-0002-6861-3343	Division of Pulmonary and Critical Care Medicine	
Ali Mahmoud	0000-0002-0441-277X	Feinberg School of Medicine	
Elizabeth S. Malsin	0000-0002-3294-6752	Division of Pulmonary and Critical Care Medicine	
Nikolay S. Markov	0000-0002-3659-4387	Division of Pulmonary and Critical Care Medicine	

Alexandra C. McQuattie-Pimentel	0000-0003-4028-9905	Pulmonary and Critical Care Medicine		
Daniel Meza	0000-0001-8154-1295	Division of Pulmonary and Critical Care Medicine		
Alexander V. Misharin	0000-0003-2879-3789	Division of Pulmonary and Critical Care Medicine		
Felix Leonardo Morales	0000-0002-2372-746X	Department of Chemical and Biological Engineering		
Luisa Morales-Nebreda	0000-0002-7126-0518	Division of Pulmonary and Critical Care Medicine		
Richard I. Morimoto	0000-0002-3431-445X	Department of Molecular Biosciences		
Ruben J. Mylvaganam	0000-0002-7203-7609	Division of Pulmonary and Critical Care Medicine		
Prasanth Nannapaneni	0000-0001-8080-3214	Northwestern Medicine Enterprise Data Warehouse		
Sophia Nozick	0000-0002-0791-6340	Division of Microbiology-Immunology		
Luís A. Nunes Amaral	0000-0002-3762-789X	Dept Chemical and Biological Engineering	Dept of Medicine	Dept. Molecular Biosciences
Egon A. Ozer	0000-0002-7131-3691	Department of Microbiology-Immunology		
Radhika Patel	0000-0002-1255-5818	Division of Pulmonary and Critical Care Medicine		
Anna E. Pawlowski	0000-0002-9047-6437	Northwestern Medicine Enterprise Data Warehouse		
Lorenzo L. Pesce	0000-0002-8015-4653	Division of Thoracic Surgery		
Chiagozie O. Pickens	0000-0002-3400-9228	Division of Pulmonary and Critical Care Medicine		
Yuliya Politanska	0000-0002-6766-988X	Division of Pulmonary and Critical Care Medicine		
Taylor A. Poor	0000-0002-2191-5037	Division of Pulmonary and Critical Care Medicine		
Michelle Hinsch Prickett	0000-0002-1119-0079	Division of Pulmonary and Critical Care Medicine		
Chao Qi	0000-0003-0741-3070	Department of Pathology		
Melissa Querrey	0000-0003-3137-8762	Division of Thoracic Surgery	Division of Pulmonary and Critical Care Medicine	
Luke V. Rasmussen	0000-0002-4497-8049	Department of Preventive Medicine		
Ziyou Ren	0000-0001-7127-4197	Division of Pulmonary and Critical Care Medicine		
Karen M. Ridge	0000-0001-7784-0014	Division of Pulmonary and Critical Care Medicine		
Madeline L Rosenbaum	0000-0001-9866-9850	Division of Pulmonary and Critical Care Medicine		
Sharon R. Rosenberg	0000-0002-0260-2545	Division of Pulmonary and Critical Care Medicine		
Timothy Rowe	0000-0003-2186-4513	Division of Pulmonary and Critical Care Medicine		
Susan R. Russell	0000-0003-3006-1018	Division of Pulmonary and Critical Care Medicine		
Marc A. Sala	0000-0002-2900-0595	Division of Pulmonary and Critical Care Medicine		
Daniel Schneider	0000-0002-3523-745X	Northwestern Medicine Enterprise Data Warehouse		
Clara J Schroedl	0000-0003-3280-4997	Division of Pulmonary and Critical Care Medicine		
Katharine Secunda	0000-0003-0310-5783	Division of Pulmonary and Critical Care Medicine		

Patrick C. Seed	0000-0001-8998-8374	Division of Pediatric Infectious Diseases	Stanley Manne Research Institute, Ann and Robert H. Lurie Children's Hospital
Karolina J. Senkow	0000-0001-9942-3529	Division of Pulmonary and Critical Care Medicine	
Todd Shamaly	0000-0003-3113-3167	NU Clinical and Translational Sciences Institute	
Elisheva D. Shanes	0000-0002-7659-7072	Department of Pathology	
Jiaxian Shen	0000-0003-4929-8955	Department of Civil & Environmental Engineering	
Ali Shilatifard	0000-0002-7490-2854	Department of Biochemistry and Molecular Genetics	
Lango Sichizya	0000-0002-1704-9471	Division of Pulmonary and Critical Care Medicine	
Lacy Simmons	0000-0003-4041-6775	Division of Infectious Disease	
Benjamin D. Singer	0000-0001-5775-8427	Division of Pulmonary and Critical Care Medicine	Department of Biochemistry and Molecular Genetics
Sean Smith	0000-0001-8796-3966	Division of Pulmonary and Critical Care Medicine	
Peter H. S. Sporn	0000-0002-1006-9437	Division of Pulmonary and Critical Care Medicine	Medical Service, Jesse Brown Veterans Affairs Medical Center
Justin Starren	0000-0002-5403-1115	Division of Health and Biomedical Informatics, Department of Preventive Medicine	
Thomas Stoeger	0000-0002-5540-4278	Department of Chemical and Biological Engineering, McCormick School of Engineering	
Jack Sumner	0000-0002-5023-8426	Department of Civil & Environmental Engineering	
Suchitra Swaminathan	0000-0002-8292-122X	Division of Rheumatology	Lurie Cancer Center; Feinberg School of Medicine
Jacob I. Sznajder	0000-0002-2089-4764	Division of Pulmonary and Critical Care Medicine	
Heliodoro Tejedor Navarro	0000-0001-5441-8101	Department of Chemical and Biological Engineering, McCormick School of Engineering	
Lindsey N. Textor	0000-0001-7699-9429	Division of Pulmonary and Critical Care Medicine	
Sanket Thakkar	0000-0002-0026-6533	Division of Thoracic Surgery	
Rade Tomic	0000-0003-2394-1287	Division of Pulmonary and Critical Care Medicine	
Betty Tran	0000-0001-6794-7088	Division of Pulmonary and Critical Care Medicine	
Kaitlyn Vitale	0000-0002-8679-6200	Pulmonary and Critical Care Medicine	
Ajay A. Wagh	0000-0003-3402-0272	Division of Pulmonary and Critical Care Medicine	
James M. Walter	0000-0001-7428-3101	Division of Pulmonary and Critical Care Medicine	
Firas Wehbe	0000-0002-3984-9584	Division of Health and Biomedical Informatics, Department of Preventive Medicine	
Deborah R. Winter	0000-0003-1806-673X	Division of Rheumatology	
Alexis Rose Wolfe	0000-0001-9321-892X	Division of Pulmonary and Critical Care Medicine	
Lisa F. Wolfe	0000-0001-9044-4147	Division of Pulmonary and Critical Care Medicine	
Richard G. Wunderink	0000-0002-8527-4195	Division of Pulmonary and Critical Care Medicine	
Anjana V. Yeldandi	0000-0001-9593-5375	Department of Pathology	
Zhan Yu	0000-0003-4389-0384	Division of Pulmonary and Critical Care Medicine	
